# Influence of age, stigma and social support on male temporary ejaculation failure on IVF oocyte retrieval day

**DOI:** 10.1186/s12958-020-00691-z

**Published:** 2021-01-13

**Authors:** Jinluan Wang, Mingyue Xue

**Affiliations:** 1 School of Traditional Chinese Medicine, Shandong University of traditional Chinese medicine, Jinan, 250014 Shandong China; 2 Center for Reproduction and Genetics of Integrated Chinese and Western Medicine, Affiliated Hospital of Shandong University of traditional Chinese medicine, Jinan, 250011 Shandong China

**Keywords:** Temporary ejaculation failure, In vitro fertilization, Age, Perceived stress, Stigma, Social support, Psychological capital, Logistic regression analysis

## Abstract

**Objective:**

To explore the social and psychological factors associated with male *Temporary Ejaculation Failure (TEF)* during In Vitro *Fertilization (IVF)*, with the goal of providing a theoretical basis for clinical intervention and treatment.

**Methods:**

The study included 75 TEF patients and 223 non-TEF patients undergoing IVF treatment at the center of reproduction and genetics of Integrated Chinese and Western medicine in the Affiliated Hospital of Shandong University of Traditional Chinese Medicine from May 2019 to May 2020. A questionnaire survey was then administered to the study subjects. The questionnaires included general information, *Perceived Stress Scale (PSS), Stigma Questionnaire, Perceived Social Support Scale (PSSS), and Positive Psychological Capital Questionnaire (PPQ)*. Logistic regression analysis was then used to analyze the social psychological factors associated with the research objectives.

**Results:**

Comparison of social demographic factors and clinical data between TEF group and non-TEF group: there were significant differences in the age and educational level between the two groups (*P*< 0.05), and the average age of the TEF group (37.01±7.11) was significantly higher than that of the non-TEF group (34.89±6.24). In addition, patients with high school or technical secondary school education levels had the lowest probability of TEF(*X*^*2*^=7.662, *P*=0.022). 2. The difference of related social and psychological factors between the two groups: the scores of perceived stress (17.57±6.51) and stigma (4.52±3.87) in the TEF group were significantly higher than those in the non-TEF group, which were (15.50±5.00, *P*< 0.05) and (2.61±3.52, *P*< 0.05), respectively. On the other hand, the scores of social support (55.31±14.04) and psychological capital (121.73±25.93) in the TEF group were significantly lower than those in the non-TEF group, which were (60.74±10.93, *P*< 0.05) and (130.31±17.32, *P*< 0.05), respectively. Results Obtained after conducting univariate logistic regression analysis indicated that age (*OR*=1.051, *P*=0.016), perceived stress (*OR*=1.073, *P*=0.005), stigma (*OR*=1.139, *P*< 0.001), family support (*OR*=0.901, *P*< 0.001), friend support (*OR*=0.932, *P*=0.023), other support (*OR*=0.915, *P*=0.004), self-efficacy (*OR*=0.947, *P*=0.009), resilience (*OR*=0.947, *P*=0.013), hope (*OR*=0.930, *P*=0.002), and optimism (*OR*=0.953, *P*=0.032) can all significantly affect male TEF.4. Moreover, the multivariate logistic regression analysis results indicated that age (*OR*=1.071, *P*=0.002) and stigma (*OR*=1.132, *P*=0.003) can positively predict TEF, while family support (*OR*=0.877, *P*=0.012) can negatively predict TEF.

**Conclusions:**

The results obtained in this study have indicated that age and stigma are independent risk factors for male TEF, while family support is a protective factor of TEF. Analyzing the treatment of TEF from a socio-psychological perspective provides a new intervention target for effectively reducing its incidence, thereby helping to improve the success rate of IVF.

## Introduction

In Vitro *Fertilization-Embryo Transfer (IVF-ET)* technology is currently one of the most widely used human assisted reproductive technologies [1]. Its success rate is affected by many factors including its own pathology factors and psychosocial factors. Previous IVF-ET studies mainly focused on female groups, and rarely discussed the impact of psychosocial factors such as perceived stress, stigma, social support, and psychological capital on males. Recently, clinical observations have found that even men with normal sexual function and semen routine examination may have temporary ejaculation problems on the day of female egg retrieval, and thus cannot successfully obtain sperm. A previous study reported that 8.3% of men among the Chinese couples who use assisted reproductive technology have *Temporary Ejaculation Failure (TEF)* on the egg retrieval day [2]. Therefore, it is increasingly becoming apparent that men who receive IVF treatment not only face economic and mental pressure but also face possible TEF. Thus, the men easily exhibit negative emotions such as anxiety and tension [3, 4]. At the same time, generation of negative emotions increases the possibility of male TEF and further reduces the success rate of IVF-ET [5]. Therefore, this study focused on male patients undergoing the IVF process, and evaluated the social and psychological factors associated with TEF.

## Materials and methods

### Research subjects

This study included 75 TEF patients and 223 non-TEF patients undergoing IVF treatment at the center of reproduction and genetics of Integrated Chinese and Western Medicine in the Affiliated Hospital of Shandong University of Traditional Chinese Medicine from May 2019 to May 2020. A questionnaire survey was conducted after the male sperm collection was completed on the day of female egg collection. The questionnaire survey included a basic information questionnaire, perceived stress scale, stigma scale, perceived social support scale, and positive psychological capital questionnaire.

### Diagnosis ,inclusion and exclusion criteria

#### Diagnosis criteria

(1) Delayed ejaculation was diagnosed in patients who had undergone sufficient sexual arousal, sexual stimulation, and hard erection for more than 30 min, but still could not complete ejaculation and thus needed medical guidance and help. (2) Insufficient ejaculation was diagnosed in patients who had a final semen volume of less than 0.5 ml (without losing any) after ejaculation was completed, and still had the ejaculation feeling and needed to collect semen again.

#### Inclusion criteria

(1) The couples have been married for more than or equal to 1 year, had normal sexual life, could ejaculate normally, did not take contraceptive measures, and underwent assisted reproduction due to female factors; (2) men who could not obtain sperm by masturbation on the day of IVF female oocyte retrieval; (3) men who had abstinence for 2–7 days; (4) the patients had no major stress events or trauma experience except infertility in recent life; (5) informed consent and voluntary participation in the survey are requested.

#### Exclusion criteria

(1) Patients with congenital malformation and dysplasia of reproductive organs; (2) patients with penile erectile dysfunction caused by trauma; (3) previous history of sexual dysfunction (erectile dysfunction, delayed ejaculation, retrograde ejaculation, and daily semen volume < 0.5 ml); (4) semen was not taken into the sterile semen cup or some semen was sprinkled outside; (5) patients with a history of mental illness and taking psychotropic drugs (including antidepressants); (6) patients with a history of chronic diseases who need to take hormone drugs and antihypertensive drugs; and (7) patients with a history of other serious diseases.

#### Research method

The research subjects were selected in strict accordance with the diagnosis and exclusion criteria. In addition, the investigators explained to all eligible research subjects the purpose, significance, and filling requirements of the questionnaires, and provided the same possible questionnaire survey environment for all subjects. After acquiring their consents, the research subjects independently filled the questionnaires to avoid interference from their wives or any other person. They were required to complete the questionnaires within 30 min and return the questionnaires on the spot. The input of original data was completed by two persons (one for input and one for supervision), followed by a double check to ensure accuracy of the data. The questionnaire consisted of the following five parts:

1. General Information Survey Form: It included sociodemographic factors and clinical disease data such as: age, Body Mass Index (BMI), residence, education level, family economy, occupation, and other sociodemographic indicators. In addition, the following clinical indicators were also acquired: marriage years, infertility duration, treatment years, with or without children, masturbation history, and relevant clinical data of the semen retrieval outcome.
2. Perceived Stress Scale (PSS): The PSS contains 10 items, and each item adopts a 5-level scoring method ranging from 0 to 4 points. The higher the score, the higher the perceived stress. The final score is used to test the patient’s perception subjective pressure [6].
3. Stigma Questionnaire: This research starts from the perception of stigma, and refers to the stigma scale for female infertility patients compiled by Fu (2015) [7]. In this study, we designed the stigma survey questionnaire administered to the TEF population. The questionnaire adopts an 8-level scoring method, ranging from 0 to 7 (never to very), with a total score ranging from 0 to 14 points. The higher the score, the stronger the stigma.
4. Perceived Social Support Scale (PSSS): This study adopted the Zimet version of PSSS revised by Zhong et al.*,* (2004) [8]. The scale has 12 items and three dimensions which include family support, friend support, and other support. The questionnaire adopts a 7-level scoring method, from 1 to 7 (strongly disagree to strongly agree), and the total score ranges from 12 to 84 points. The higher the score, the higher the PSSS.
5. Positive Psychological Capital Questionnaire (PPQ): Psychological capital refers to the positive mental state that an individual exhibits during growth. This study adopted a questionnaire compiled by Zhong et al.*,* (2010) [9]. In total, the questionnaire contains 26 items and uses a 7-level scoring method. The total score ranges from 26 to182 points.

### Statistical analysis

The SPSS 19.0 software was used for statistical analysis. Independent sample t-test and chi-square test were used to analyze whether the scores and differences of social demographic factors, clinical data, perceived pressure, stigma, social support, and psychological capital of patients with TEF were statistically significant (*P*< 0.05). Furthermore, logistic regression was used to clarify the independent influencing factors of TEF.

## Results

### Comparison of sociodemographic factors and clinical data between the two groups

The obtained results indicated that the age and educational level of the two groups were statistically significant (*P*< 0.05). The average age of the TEF group (37.01±7.11) was significantly higher than that of the non-TEF group (34.89±6.24), while the ejaculation success rate of males with a high school or technical secondary school education was relatively higher. However, there was no statistical difference between the two in the other demographic characteristics and clinical data (*P*> 0.05). The obtained results are shown in Table 1.

**Table 1:** Comparison of sociodemographic factors and clinical data between the two groups

Item	TEF Group(***n***=75)	Non-TEF Group(***n***=223)	***t /X***^***2***^	***P value***
**Age**	37.01±7.11	34.89±6.24^a^	−2.457	0.015
**Body Mass Index**				
< 25 kg/m^2^	37 (49.3%)	104 (46.6%)	0.164	0.686
≥25 kg/m^2^	38 (50.7%)	119 (53.4%)		
**Marriage Life**				
≤5 years	35 (46.7%)	104 (46.6%)	0.000	0.996
> 5 years	40 (53.3%)	119 (53.4%)		
**Residence**				
Urban	52 (69.3%)	155 (69.5%)	0.001	0.977
Rural	23 (30.7%)	68 (30.5%)		
**Education Level**				
Junior high school and below	19 (25.3%)	47 (21.1%)^a^	7.662	0.022
High school or technical secondary school	8 (10.7%)	58 (26.0%)^a^		
College degree and above	48 (64.0%)	118 (52.9%)^a^		
**Family Economic Conditions**				
Income is less than expenditure	9 (12.0%)	16 (7.2%)	2.245	0.325
Balance of income and expenditure	44 (58.7%)	148 (66.4%)		
Income is more than expenditure	22 (29.3%)	59 (26.4%)		
**Have children or not**				
Have children	31 (41.3%)	89 (39.9%)	0.047	0.828
Have no children	44 (58.7%)	134 (60.1%)		
**Infertility Duration**				
< 3 years	26 (34.7%)	76 (34.1%)	0.072	0.964
3~5 years	29 (38.7%)	90 (40.4%)		
> 5 years	20 (26.6%)	57 (25.5%)		
**Treatment Duration**				
< 2 years	25 (33.3%)	79 (35.4%)	3.963	0.265
2~5 years	37 (49.4%)	111 (49.8%)		
> 5 years	13 (17.3%)	33 (14.8%)		
**Masturbation history**				
Never	17 (22.7%)	53 (23.8%)	0.038	0.846
Have at least once	58 (77.3%)	170 (76.2%)		

Values given as number (percent). Age in years

^a^Compared with TEF Group, *P< 0.05*

### Comparison of the psychosocial factors for the two groups

The obtained results indicated that perceived stress, stigma, social support, and psychological capital were significantly different between the TEF group and the non-TEF group (*P* < 0.05). The TEF group had higher scores for perceived stress and stigma than the non-TEF group. In addition, the TEF group’s scores in the three dimensions of social support including family support (18.83±5.71), friend support (18.56±4.73), and other support (17.92±5.09) were lower than those of the non-TEF group, which were (21.25±4.29), (19.89±4.18), and (19.60±3.99), respectively. Moreover, the TEF group had higher scores for psychological capital including self-efficacy (31.91±7.64), resilience (29.27±7.58), hope (30.01±6.94), and optimism (30.55±7.41) than those of the non-TEF group, which were (34.21±6.03), (31.38±5.73), (32.46±5.25), and (32.27±5.32), respectively. The obtained results are shown in Table 2.

**Table 2:** Comparison of the psychosocial factors for the two groups

Item	TEF Group(***n***=75)($\overline{x}$*±s*)	Non-TEF Group(***n***=223)($\overline{x}$*±s*)	***t***	***P value***
**Perceived stress**	17.57±6.51	15.50±5.00	−2.870	0.004
**Stigma**	4.52±3.87	2.61±3.52	−3.973	< 0.001
**Social Support**	55.31±14.04	60.74±10.93	3.457	0.001
**Family Support**	18.83±5.71	21.25±4.29	3.877	< 0.001
**Friend Support**	18.56±4.73	19.89±4.18	2.308	0.022
**Other Supports**	17.92±5.09	19.60±3.99	2.936	0.004
**Psychological capital**	121.73±25.93	130.31±17.32	3.240	0.001
**Self-efficacy**	31.91±7.64	34.21±6.03	2.664	0.008
**Resilience**	29.27±7.58	31.38±5.73	2.532	0.012
**Hope**	30.01±6.94	32.46±5.25	3.201	0.002
**Optimism**	30.55±7.41	32.27±5.32	2.182	0.030

Values given as mean±standard deviation

### Single factor logistic regression analysis of TEF predictors

The regression equation for single factor logistic regression analysis used the occurrence of TEF as the dependent variable (0 = non TEF, 1= TEF), while the three dimensions of age, education level, perceived pressure, stigma, family support, friend support, and other support in social support, and four dimensions of self-efficacy, resilience, hope, and optimism in psychological capital were taken as independent variables. The obtained results indicated that age, perceived stress, stigma, family support, friend support, other support, self-efficacy, resilience, hope, and optimism can significantly affect male TEF. Among them, age, perceived stress, and stigma can positively predict the occurrence of TEF because they are the risk factors of TEF. On the other hand, family support, friend support, other support, self-efficacy, resilience, hope and optimism were the protective factors of TEF because they were able to negatively predict the occurrence of TEF. The obtained results are shown in Table 3 (only meaningful predictive variables are shown in the table).

**Table 3:** Single factor logistic regression analysis of TEF predictors

Item	B	S.E.	Wald	***P***	OR	95%CI
**Age**	0.049	0.020	5.845	0.016	1.051	(1.009,1.094)
**Perceived stress**	0.070	0.025	7.786	0.005	1.073	(1.021,1.127)
**Stigma**	0.130	0.035	14.086	< 0.001	1.139	(1.064,1.219)
**Social support**						
**Family support**	−0.105	0.028	13.535	< 0.001	0.901	(0.852,0.952)
**Friend support**	−0.071	0.031	5.159	0.023	0.932	(0.877,0.990)
**Other support**	−0.089	0.031	8.144	0.004	0.915	(0.860,0.972)
**Psychological capital**						
**Self-efficacy**	−0.055	0.021	6.808	0.009	0.947	(0.909,0.986)
**Resilience**	−0.055	0.022	6.164	0.013	0.947	(0.907,0.989)
**Hope**	−0.072	0.023	9.571	0.002	0.930	(0.889,0.974)
**Optimism**	−0.048	0.022	4.620	0.032	0.953	(0.913,0.996)

### Multivariate logistic regression analysis of TEF predictors

The statistical indicators in the above univariate analysis were included in the multivariate logistic regression analysis in order to further analyze the joint effects of the various factors of TEF. The obtained results indicated that age, stigma, and family support can significantly affect men’s TEF under the joint action of multiple factors. Age and stigma are the independent risk factors for the occurrence of TEF because they can positively predict TEF, while family support is an independent protective factor for the occurrence of TEF because it can negatively predict TEF occurrence. The obtained results are shown in Table 4.

**Table 4:** Multivariate logistic regression analysis of TEF predictors

Item	B	S.E.	Wald	***P***	OR	95%CI
**Age**	0.068	0.022	9.296	0.002	1.071	(1.025, 1.119)
**Perceived stress**	0.002	0.003	0.004	0.951	1.002	(0.940, 1.068)
**Stigma**	0.124	0.041	9.108	0.003	1.132	(1.044, 1.227)
**Social support**						
**Family support**	−0.132	0.052	6.342	0.012	0.877	(0.791, 0.971)
**Friend support**	0.061	0.049	1.509	0.219	1.063	(0.965, 1.170)
**Other support**	0.005	0.052	0.008	0.928	1.005	(0.907, 1.114)
**Psychological capital**						
**Self-efficacy**	−0.014	0.032	0.189	0.664	0.986	(0.926, 1.051)
**Resilience**	0.012	0.030	0.163	0.686	1.012	(0.955, 1.073)
**Hope**	−0.069	0.045	2.396	0.122	0.933	(0.854, 1.019)
**Optimism**	0.039	0.041	0.937	0.333	1.040	(0.960, 1.126)

## Discussions

The IVF-ET treatment process is extremely complicated and requires efficient coordination of multiple links. Any small mistake may lead to pregnancy failure. Therefore, smooth collection of sperm by men on the day of female oocyte retrieval is a vital part of the IVF treatment process. This study analyzed the relationship between the occurrence of male TEF and social psychological factors during the process of IVF. The main aim of the study was to lay a foundation for targeted psychological intervention, and to reduce the risk of male TEF in IVF treatment, thereby significantly reducing its adverse impact on the success rate of IVF-ET.

### The influence of sociodemographic factors on TEF

The results obtained in this study indicated that the age of men in the TEF group was higher than those in the non-TEF group, and age is an independent risk factor for TEF. This result was consistent with the results reported by Shang (2011) [10]. The level of androgen and sexual desire in men gradually decreased with the increase of age, while the threshold for sexual organ stimulation gradually rises. Therefore, it is difficult to induce ejaculation by general sexual stimulation. This result suggests that older men are more likely to have TEF. At the same time, the study found that men with higher or lower education were more likely to have TEF. Li et al.*,* (2020) reported that there is a certain correlation between the level of male education and the occurrence of ejaculation disorder (*OR*=0.894) [11]. In addition, Glina (2005) [12] and other studies have shown that men with a higher education are more likely to have TEF. This can be attributed to the fact that men with higher education know how to collect information related to the treatment, but they are prone to producing more negative emotions such as anxiety and tension due to over misinterpreting the relevant data. On the other hand, men with a lower education level have a poor ability of collecting relevant treatment information, and they are more likely to be nervous because they do not understand the treatment process.

### The impact of perceived stress on TEF

Univariate logistic regression analysis results indicated that perceived stress could positively predict TEF. In the process of IVF treatment, men face multiple pressures such as economic and psychological. Their perceived pressure is at a high level, and thus they are prone to producing more negative emotions. These negative emotions may stimulate the secretion of prolactin thereby reducing their sexual desire. In addition, further multivariate regression analysis showed that perceived stress had no significant predictive effect on TEF. Some studies have reported that there is a significant negative correlation between perceived stress and social support [13]. Therefore, in multivariate regression analysis, perceived stress may not be able to predict the occurrence of TEF due to the influence of “social support”.

### The impact of stigma on TEF

The results obtained after univariate and multivariate logistic regression analyses indicated that TEF, which was positively predicted by stigma, was an independent risk factor for TEF. Infertility patients are prone to feeling an inferiority complex, shame, and discrimination because they are affected by their own diseases and the traditional Chinese ideology of “three kinds of filial piety, no offspring is the most important” [14, 15]. Gana (2016) reported that the emergence of negative emotions causes a significant decrease in male sexual desire, which most likely leads to TEF [5]. Although stigma has a certain impact on male ejaculation, sufficient social support and strong psychological capital can offset part of the negative psychology and reduce the incidence of TEF [16].

### The impact of social support on TEF

Results obtained after univariate logistic regression analysis indicated that the three dimensions of social support can negatively predict the occurrence of TEF, which is consistent with the results reported by Qadir (2015) [17]. Several studies have confirmed that social support is a protective external resource. One previous study reported that the negative emotions of an individual will be significantly reduced if the individual receives emotional and material support from his family and friends when facing difficult events [18]. Further multivariate regression analysis done in this study showed that only family support was the independent protective factor of TEF. In the process of IVF clinical treatment, some wives can result to blames and complains, and do not give support and encouragement for men’s sperm extraction. At the same time, sexual life is no longer an intimate way between husband and wife, but is only a means of procreation. Therefore, men are prone to TEF during the sperm extraction process. Several studies have reported that poor family support can cause certain effects on male sperm extraction [19–21]. During the process of treatment, the medical staff can take the initiative to communicate effectively with the couples receiving treatment, fully understand their current problems, encourage, and inform men and their wives on the importance of social support, especially family support. This can help men feel the power of family support thereby improving the success rate of sperm extraction.

### The impact of psychological capital on TEF

Univariate Logistic regression analysis results indicated that the four dimensions of psychological capital could negatively predict the occurrence of TEF. However, further multivariate regression analysis showed that the four dimensions did not significantly predict the occurrence of TEF. This can be attributed to a certain correlation between psychological capital and family support. A previous study reported that there is a significant positive correlation between psychological capital and family support [22]. Therefore, prediction of the psychological capital on TEF will be affected in the multi factor regression analysis.

The limitation of this study is that the patients are all from one single hospital in China and there is a lack of large sample data statistics in many regions and centers, so the representativeness and generalization of the results are limited. The study uses self-rating scales to assess individuals’ perceived stress, stigma, social support, and psychological capital levels, and there may be reporting bias. However, from the perspective of positive psychology, this study explores the influencing factors of TEF in IVF treatment and overcomes the limitation of previous studies on negative psychological factors, which is a beneficial supplement to the current research.

## Conclusions

Infertility has had many impacts on the marriage emotion and work life for both men and women, and serious cases might lead to psychological problems. The negative psychological emotions significantly affect the treatment effect for a couple and aggravates the infertility problem [23]. This study analyzed the related social and psychological factors that led to the occurrence of male TEF during IVF. The results obtained in this study have indicated that age and stigma are independent risk factors for male TEF, while family support is a protective factor of TEF. The results obtained in this study can be applied for the clinical prediction of male TEF on the day of female oocyte retrieval, thereby providing a new intervention target for effectively reducing the incidence of TEF. In addition, they can provide theoretical support for making targeted and effective psychological intervention measures, thereby improving the success rate of IVF-ET.

## Data Availability

All data generated or analyzed during this study are included in this published article.

## Abbreviations

IVF: In Vitro Fertilization
TEF: Temporary Ejaculation Failure
IVF-ET: In Vitro Fertilization-Embryo Transfer
PSS: Perceived Stress Scale
PSSS: Perceived Social Support Scale
PPQ: Positive Psychological Capital Questionnaire
BMI: Body Mass Index
